# State of the Faculty and of Physic in Algiers

**Published:** 1840-06

**Authors:** 


*State of the Faculty and of Physic in Algiers.*—Physic would seem to be at a low ebb in Algiers. The doctors certainly are so. The following extract from an extract in the Medical Gazette will exhibit the state of things in that country.

"Dr. Bohn first introduced vaccination, and practised it in the family of the deposed Dey himself, who, however, did not give him a princely fee ; and, generally speaking, people are here unwilling to give aught to physician or apothecary.

As to the native physicians, the Dey had a kind of protomedicus, who decided medico-legal questions, and created other physicians for a few piasters, without being exactly able to read or write. If a man was able to shave well, if he could compound a plaster, and cure a hurt, he bought the privilege, and prescribed at his own pleasure the whole contents of any of the six Moorish apothecaries' shops ; bark with or without theriaca at all times ; and in all fevers, opium, sarsaparilla, calomel, pimento, cantharides, and opodeldoch. Ismael Ben Mehmed enjoyed the greatest share of public confidence ; he gave Dr. Schonberg an extract from the Arabic work of Ben Huesina, who lived 700 years ago, and a catalogue of his own drugs. His shop, the largest in the town, contained 70 jars, 30 bottles, 20 boxes and several drawers. He obtained medicines from abroad, prepared others himself, and possesses a still and retort. He is afraid of mercury against syphilis, and thinks he can do without it.

Ismael Ben Mehmed is acquainted with remittent and intermittent fevers, and their varieties. His surgical apparatus consisted of a common case of dressing instruments."

*Medico-Chirurgical Review.*

